# Correction to “Structural Effects of FeN_4_ Active Sites Surrounded by Fourteen-Membered Ring Ligands on Oxygen Reduction Reaction Activity and Durability”

**DOI:** 10.1021/acscatal.5c00817

**Published:** 2025-02-14

**Authors:** Zhiqing Feng, Soutaro Honda, Junya Ohyama, Yasushi Iwata, Keisuke Awaya, Hiroshi Yoshida, Masato Machida, Kotaro Higashi, Tomoya Uruga, Naomi Kawamura, Ryota Goto, Takeo Ichihara, Ryoichi Kojima, Makoto Moriya, Hideo Notsu, Shinsuke Nagata, Mami Miyoshi, Teruaki Hayakawa, Yuta Nabae

In Figure 5(b), the labels “Model A” and “Model B” were mistakenly swapped. The corrected version of Figure 5 is displayed below.

**Figure 5:**
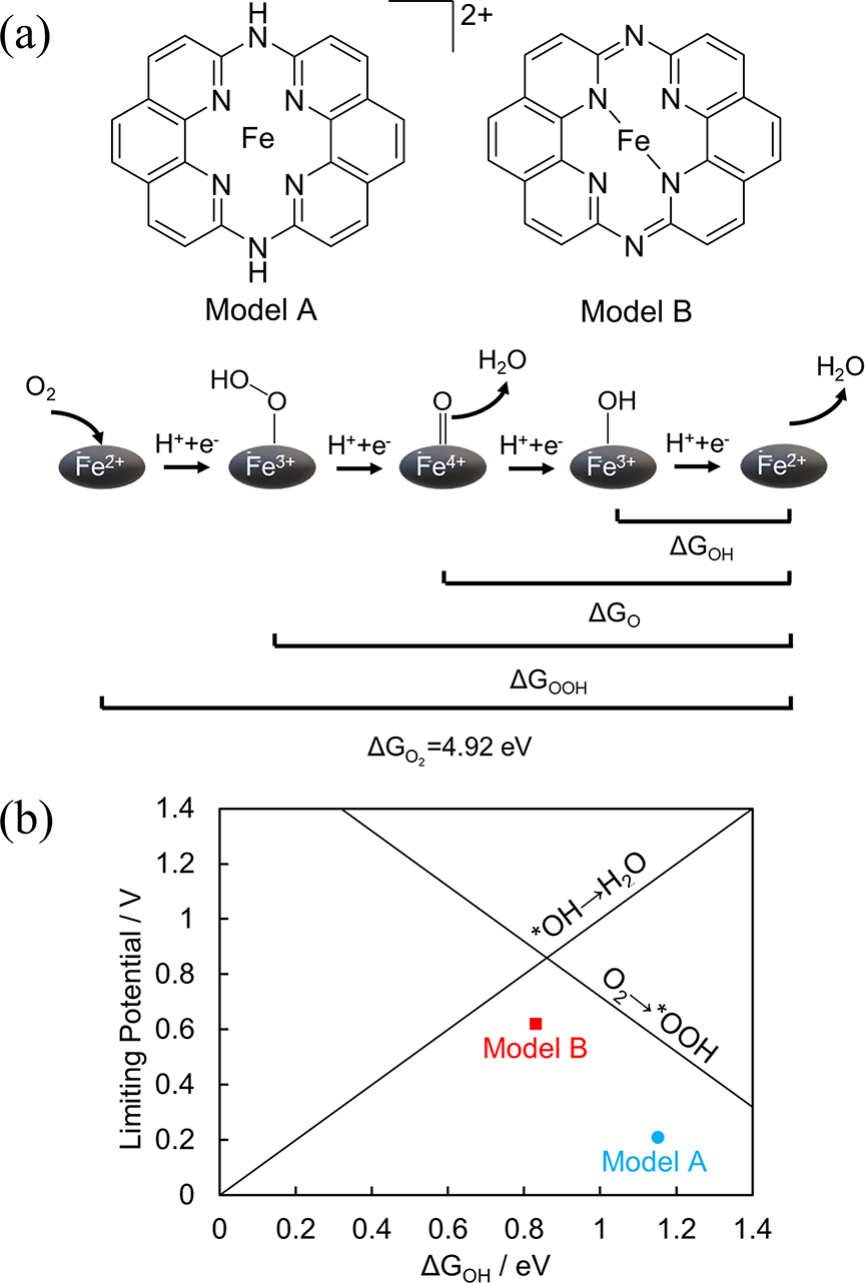
(a) Models and ORR scheme considered in the DFT simulation. (b) Relationship between the limiting potential and Δ*G*_OH_ values for Models A and B overlaying a volcano-type plot drawn based on previous theoretical calculations.

